# Multimorbidity and functioning disparities in retired older adults: longitudinal trajectories, disease clusters, and mediating mechanisms

**DOI:** 10.1186/s12877-026-07388-9

**Published:** 2026-04-06

**Authors:** Nnaelue Godfrey Ojijieme, Long Xiao

**Affiliations:** 1 Faculty of Economics and Management, Xi’an Fanyi University, Xi’an, 710105 China; 2 School of Politics, Law and Public Administration, Yan’an University, Yan’an, 716000, China

**Keywords:** Functioning, Multimorbidity, Body Mass Index

## Abstract

**Background:**

Multimorbidity is a major determinant of older adults’ functioning, yet its long-term pathways and underlying mechanisms remain insufficiently understood. Retired older adults face unique vulnerabilities due to declining occupational activity, reduced socioeconomic buffers, and increased dependence on functional capacity. This study examines how multimorbidity shapes functioning over sixteen years and whether Body Mass Index (BMI) mediates these association functioning and well-being in functioning and well-being in .

**Methods:**

Data from older adults aged 60 and over, in the Health and Retirement Study (2004–2020) were analyzed using growth curve models to estimate baseline levels and rates of change in functioning (*N* = 1,238), mixed-effects models to assess disease cluster, sex and race effects (*N* = 1,238), and conditional process analyses to test BMI mediation (*N* = 8,654).

**Results:**

Multimorbidity is associated with reduced baseline functioning in the physical functioning and disability domains, but did not accelerate decline across all domains, indicating that the principal disadvantage emerges early and persists over time. Mixed-effects models showed strong negative and domain-specific associations, with musculoskeletal conditions most strongly associated with impairing physical functioning and respiratory conditions exerting the greatest association with disability. Significant sex and racial disparities were observed, with females and minority groups exhibiting consistently poorer functioning. BMI partially mediated the effects of multimorbidity on physical and cognitive functioning, with indirect effects strengthening modestly over the baseline and terminal periods. However, the disability domain was not mediated, suggesting that disability reflects more entrenched functional losses.

**Conclusions:**

Multimorbidity shapes functioning primarily through early deficits and disease cluster-specific pathways. BMI represents a modifiable mechanism that can enhance physical and cognitive functioning, supporting interventions focused on weight management, tailored chronic-disease care, and equity-centered strategies to preserve functioning and promote healthy aging among retired older adults.

**Supplementary Information:**

The online version contains supplementary material available at 10.1186/s12877-026-07388-9.

## Introduction

Population aging is transforming health landscapes worldwide, intensifying the challenge of preserving functioning and well-being in later life [1–6]. Among the most pervasive barriers to older adults’ functioning is multimorbidity, the co-existence of two or more chronic conditions, which complicates clinical management and is associated with accelerated losses in physical capacity, increased disability, and higher healthcare utilization [7–9]. In the United States, where the older adult population is substantial and growing, multimorbidity contributes to mounting social and economic burdens, as well as reductions in subjective well-being and life satisfaction [10, 11].

Functioning in older adults can be meaningfully evaluated across three interrelated domains — disability, physical functioning, and cognitive functioning, following an extended interpretation of Rowe and Kahn’s model [12]. Disability reflects the ability to perform Activities of Daily Living (ADL) and Instrumental Activities of Daily Living (IADL), such as walking across a room, managing finances, shopping, and preparing meals [13, 14]. Physical functioning captures mobility-intensive activities requiring muscle use, including walking long distances, ascending stairs, rising from a chair, crouching, and lifting objects [14, 15]. Cognitive functioning is commonly assessed using composite measures such as the Total Cognition Score, incorporating memory recall, mental status, and executive function [16, 17].

A substantial body of research has examined how multimorbidity affects these specific functional domains. Older adults with higher disease burden consistently show lower capability in ADL/IADL tasks and physical activities, with multimorbidity increasing both the likelihood of incident disability [9, 18, 19]. Evidence for cognitive outcomes is more mixed: some studies suggest multimorbidity, particularly cardiometabolic and neurological conditions, negatively impacts cognition, while others find weaker or no associations [10, 20]. Much of this literature is cross-sectional, highlighting associations between higher disease burden and poorer functioning across disability, physical functioning, and cognition. Longitudinal studies further indicate that multimorbidity predicts steeper declines in some functional domains, as well as higher risks of institutionalization, healthcare utilization, and mortality [8, 10, 18, 19, 21–26].

Recognizing that the health consequences of multimorbidity depend not only on the number of conditions but also on their composition, researchers increasingly use disease cluster approaches which reveal that certain clusters disproportionately affect older adults’ health [27, 28]. Clustering conditions into clinically meaningful groups, such as cardiometabolic, musculoskeletal, neurological, and respiratory conditions, reveals distinct domain-specific risk profiles, enhances clinical relevance, and helps identify combinations of conditions that may benefit from targeted prevention and rehabilitation strategies.

Despite these advances, some methodological and substantive gaps persist. Firstly, a large portion of the literature remains cross-sectional or relies on simple disease counts, which obscures the timing and heterogeneity of multimorbidity’s effects. Cross-sectional associations cannot distinguish whether multimorbidity precipitates health decline, reflects accumulated lifetime disadvantage, or is confounded by earlier life exposures. Secondly, although some longitudinal studies estimate average change, few explicitly disaggregate multimorbidity’s influence on baseline functional status (intercepts) versus rates of change (slopes) across extended follow-up periods. Disentangling intercepts from slopes is essential because early baseline deficits and accelerated decline imply different prevention and intervention windows. Thirdly, the mechanistic pathways linking multimorbidity to functioning are under-examined: while factors such as inflammation, polypharmacy, and social determinants have been proposed, relatively few studies have evaluated modifiable physiological mediators (e.g., Body Mass Index [BMI]) that could enhance interventions [29–31].

BMI is a particularly promising but underutilized mediator in multimorbidity research. Prior work documents an ambivalent relationship between adiposity and late-life outcomes: higher BMI is sometimes associated with better short-term survival or cognitive outcomes (the so-called “obesity paradox”) while also contributing to metabolic risk, inflammation, and functional impairment over time [32–34]. Investigating BMI as a mediating pathway can clarify whether and how weight status transmits or modifies the functional burden of multimorbidity, and whether its role differs across functional domains such as cognition, physical functioning, and disability.

A further important gap concerns population focus. Most multimorbidity studies examine community-dwelling older adults as a broad group, whereas retired older adults represent a unique demographic of high policy relevance. Retirement is a major life transition associated with reduced income, lower social participation, shifts in daily structure, and changes in health behaviors [35, 36]. Moreover, retired adults typically experience rapid increases in multimorbidity, declines in physical functioning, and increased healthcare dependency [37]. Studying this population is therefore crucial for designing targeted interventions, planning post-retirement health policies, and strengthening aging-in-place strategies. As functional ability strongly predicts independence, institutionalization, and quality of life, understanding multimorbidity patterns specifically in retired older adults provides insight into a group facing distinctive vulnerabilities and care needs.

Furthermore, prior research has often neglected how multimorbidity’s functional consequences vary across social strata. Evidence suggests sex and race influence many health processes in aging, with males potentially being physically stronger and having fewer disabilities [38, 39]. Yet few studies have systematically examined whether the adverse effects of multimorbidity on older adults’ functioning differ by sex and racial group in US samples. This omission limits translational potential and risks perpetuating monolithic recommendations that may widen rather than narrow health disparities.

To address these gaps, this study adopts an integrative, multi-method analytic framework that combines (a) growth curve modeling, (b) mixed-effects modeling, and (c) conditional process (mediation) analysis. Growth curve models allow explicit estimation of both intercepts and slopes of functioning across multiple waves, providing a test of whether multimorbidity predicts lower baseline functioning, its decline rate, or both. These functioning were independently assessed across disability, cognitive and physical functioning domains. Mixed-effects models then unpack heterogeneity by estimating the domain-specific impacts of five clinically meaningful clusters: cardiometabolic, neurological, respiratory, musculoskeletal, and cancer conditions, acknowledging that the health consequences of multimorbidity depend on disease composition rather than simple counts. Finally, conditional process analysis examines whether BMI mediates relationships between multimorbidity and functioning, offering mechanistic insight into modifiable pathways suggested by prior literature [40–42].

By triangulating these complementary approaches within a longitudinal US sample, this study advances the field in three main ways. First, it clarifies how disease accumulation shapes both the starting point and the pace of decline in functioning, distinguishing early deficits from accelerated deterioration, an issue especially salient for retired older adults, who often experience shifts in daily routines, reduced occupational physical activity, and increased reliance on personal functional capacity during post-retirement years. Second, it illuminates which disease clusters pose the greatest risks to specific functional domains and whether these patterns vary by sex and race, offering insights particularly relevant to retired individuals whose health disparities tend to widen after labor-force exit due to differences in lifetime work conditions, socioeconomic resources, and access to care. Third, it identifies BMI as a potential intervention target, estimating its mediating role across multiple functional domains and over time, critical for retired adults who face heightened risk of weight dysregulation linked to reduced mobility, medication use, and changes in dietary habits. Collectively, these contributions provide empirical insight into theoretical models of cumulative advantage/disadvantage^1^ [43, 44] by illustrating how the accumulation of chronic conditions and related physiological factors, such as BMI, may shape disparities in functional outcomes in later life. At the same time, the study enhances methodological precision and offers actionable evidence for clinical and public-health strategies aimed at preserving functioning and promoting healthy aging among retired older adults.

## Methods

### Research design

#### Research framework and content

The data for this analysis derives from the US Health and Retirement Study (HRS). The HRS is essential for national comparisons on aging, capturing diverse health, economic, and social indicators that allow for a nuanced analysis of aging trends and policy implications in target countries. The HRS is a biennial survey of over 37,000 Americans aged 50 and older, covering 23,000 households (and is nationally representative). HRS collects data on several aspects of aging, focusing on health, income, wealth, cognition, family dynamics, and healthcare services. The study’s multidisciplinary approach facilitates a comprehensive understanding of aging's impact on economic and social factors [45]. Following precedents from studies on retired populations aged 60 and above [46], We included 1238 individuals who were “completely retired” from the base to the terminal period (2004 to 2020) for the growth curve and mixed effects modeling. Because the mediation analysis is cross-sectional, 8654 and 7941 completely retired older adults at baseline (2004) and terminal period (2020) are utilized. These samples differed significantly because while the first analysis captured individuals that were retired at baseline and remained retired till the terminal period (hence, a smaller total sample), the mediation analysis only captured individuals that were retired at baseline and terminal period.

**Fig. 1:**
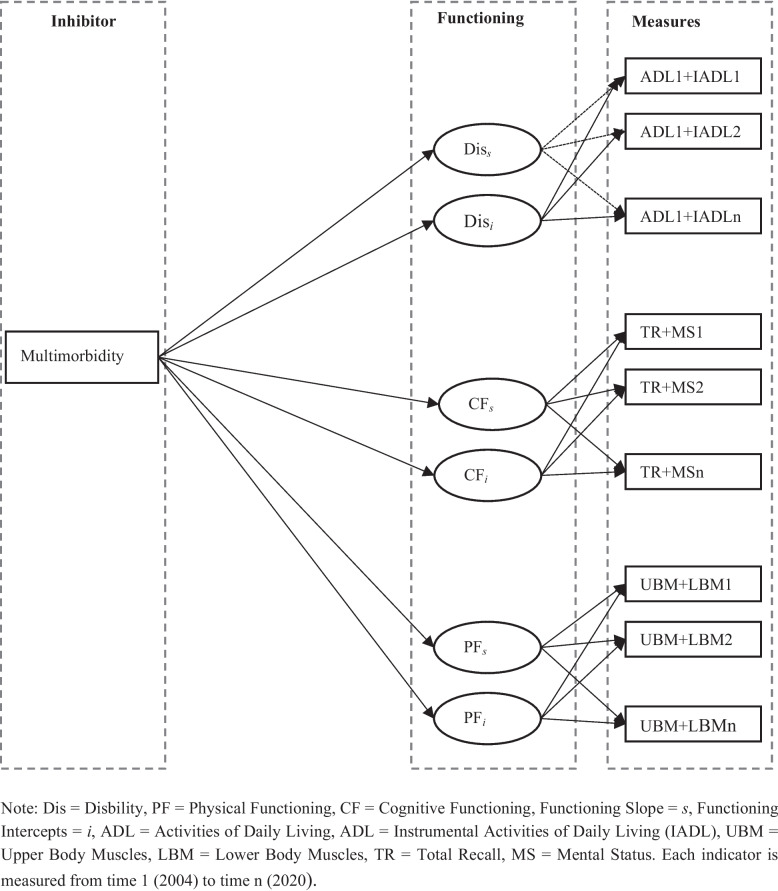
Analytical framework for the effects of multimorbidity on functioning trajectories

**Fig. 2:**
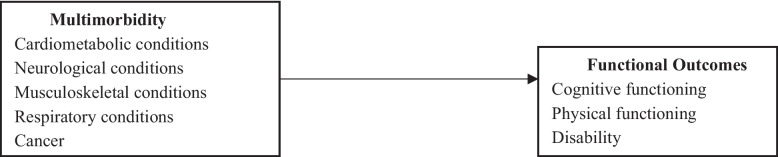
Analytical framework for the effects of multimorbidity on older adults’ functioning

**Fig. 3:**
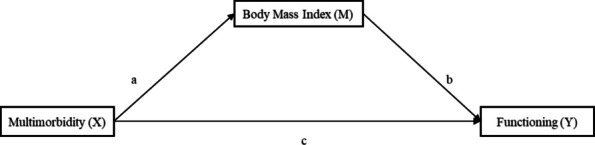
Conceptual framework of the mediating influence of BMI on the association between multimorbidity and functioning

Figures 1, 2 and 3 represent the analysis frameworks for the relationships examined in this research. Figure 1 shows how multimorbidity affects the trajectory of functioning over time, specifically the intercept (baseline level) and slope (rate of decline). This longitudinal perspective is critical because it allows the analysis to distinguish whether multimorbidity primarily associated with depressed initial levels of functioning, accelerates decline over time, or both. Figure 2 illustrates how multimorbidity, as disaggregated into five chronic disease clusters (cardiometabolic, neurological, respiratory, musculoskeletal, and cancer conditions), influences the three core functional outcomes of — cognitive functioning, physical functioning, and disability. By disaggregating multimorbidity into these clusters, the framework provides a nuanced view of which types of chronic conditions exert the strongest influence on different functioning facets, thereby offering an evidence-based foundation for tailored interventions. Figure 3 assesses the mediating capacity of BMI in regulating the effect of multimorbidity on the three older adults’ functional outcomes. This adds to the body of literature deciphering additional pathways to mitigate the adverse effects of multimorbidity, including enhancing older adults’ functioning and independence.

The use of these three analytical approaches provides a more comprehensive assessment of how multimorbidity shapes functioning in later life. Growth curve modeling captures baseline levels and trajectories of functional change over time, allowing the study to distinguish between initial disadvantages and subsequent rates of decline. Mixed-effects models complement this approach by examining the domain-specific effects of multimorbidity clusters and assessing disparities across key demographic groups, particularly sex and race, which are central to understanding health inequalities in the United States. Finally, mediation analysis evaluates BMI as a potential physiological pathway linking multimorbidity to functional outcomes. Taken together, these approaches enable the study to examine the timing, heterogeneity, and mechanisms of multimorbidity’s impact, thereby identifying not only who may be most vulnerable but also when and through which pathways interventions may be most effective.

Beyond their methodological advantages, these frameworks also carry significant implications for theory and practice. Theoretically, they provide more insight into the cumulative disadvantage model by showing how chronic conditions interact with social, behavioral, biologic and demographic factors to shape older adults’ functioning. This approach strengthens the evidence base for life-course theories of health, which emphasize the compounding effects of disadvantage and disease accumulation over time. Practically, the findings offer actionable guidance for policymakers, healthcare providers, and public health practitioners in the US. For instance, identifying which disease clusters drive the steepest declines in functioning can inform priority-setting in preventive care, chronic disease management, and social support programs.

#### Variable description

Functioning is evaluated across three primary domains: disability, physical functioning, and cognitive functioning, based on an extended interpretation of Rowe and Kahn's model by McLaughlin et al. [12]. Disability assesses the capability of retired older adults to execute Activities of Daily Living activities (ADL) and Instrumental Activities of Daily Living activities (IADL). It encompasses ten activities that include walking across a room, handling finances, shopping, and meal preparation. The scores for each activity are reverse-coded and subsequently aggregated to provide a composite score ranging from 0 (showing an inability to execute any activities) to 10 (representing full capability). Individuals attaining a score of 9 or above is designated as possessing "high ability," a criterion derived from the parent model McLaughlin et al. [12], as nearly no participants obtained a perfect score [13–15]. Physical functioning measures the capacity of older adults to do mobility-intensive activities necessitating the use of muscle groups. This encompasses nine distinct activities: "walking many blocks, walking one block, traversing a room, ascending flights of stairs, sitting for prolonged durations, rising from a chair, crouching, and manipulating huge things." The score for each activity is reverse-coded and aggregated to get a composite score ranging from 0 (poor functioning) to 9 (high functioning). A score of 8 or above in this area signifies high physical functioning [14, 15]. The cognitive functioning domain is assessed by the Total Cognition Score. Total cognition score is an ordinal outcome (ranging from 0 to 35 and is the sum of the weighted composite scores of the total recall and mental status indices — word recall (range 0—20,serial 7 subtraction (range 0—5; backward counting (range 0—2; objects (range 0—2, date (range 0—4, and president/vice president recognition (range 0—2. A score of 22 or above denotes robust cognitive health [14, 16, 17].

Multimorbidity – Participants indicated doctor’s diagnoses for eight chronic conditions.^2^ Based on pertinent studies, we classified these diseases into five multimorbidity categories for the sex and race stratifies analysis: cardiometabolic (diabetes, heart problems), neurological (hypertension, stroke, and psychiatric problems), musculoskeletal (arthritis), respiratory (chronic lung disease), and cancer [27, 28]. For trajectory and mediation analysis, multimorbidity is defined as the existence of a doctor's diagnosis of two or more chronic conditions in a single individual, based on a relevant study [8]. Older adults with none or one chronic condition are coded as 0, while older adults with two or more chronic conditions are coded as 1. While Body Mass Index (BMI) is a continuous variable is calculated as weight divided by the square of height. Height is converted into meters and weight into kilograms and ranges from 12.6 to 58.6.

The covariates in this study are derived from similar studies and are summarized in Tables 1 and 2. “Age is entered as a continuous variable ranging from 60 to 104 years old. We coded sex as 1 = female and 2 = male. Race ranged from 1 = white/Caucasian, 2 = black/African American, 3 = other. We coded marital status as 1 = “married, married (spouse absent), partnered, and 0 = separated, divorced, widowed, and never married, separated, and divorced.” Educational attainment is entered as 1 = less than high school, 2 = GED, 3 = high school graduate, 4 = some college, 5 = college and above. Based on Amadeo [47], socioeconomic status is calculated as the sum of total assets (less debt) and is coded as (−141,999 to 6030 = 0) poverty class, (6030.1 to 42,000 = 1) lower-middle class, (42,001 to 104,000 = 2) middle class, (104,001 to 201,000 = 3) upper middle class, (201,001 to 608,000 = 4) wealthy, and (608,001 and above = 5) super wealthy. Smoking status = 0 for non-smokers and 1 for smokers. Drinking frequency ranged from 0 = none to 7 = daily drinking” [10].

**Table 1:** Sample sociodemographic characteristics based on long data format

**Variables**		Waves		
	2004	2018	2020	Total
N	1,238 (11.1%)	1,238 (11.1%)	1,238 (11.1%)	11,142 (100.0%)
***Functioning Facets***				
*Cognition Functioning*	23.865 (4.010)	21.310 (4.937)	20.628 (5.098)	22.444 (4.549)
*Disability*				
0	0 (0.0%)	4 (0.3%)	9 (0.7%)	17 (0.2%)
1	0 (0.0%)	7 (0.6%)	9 (0.7%)	22 (0.2%)
2	0 (0.0%)	7 (0.6%)	15 (1.2%)	39 (0.4%)
3	6 (0.5%)	15 (1.2%)	32 (2.6%)	79 (0.7%)
4	7 (0.6%)	24 (1.9%)	26 (2.1%)	104 (0.9%)
5	8 (0.6%)	48 (3.9%)	53 (4.3%)	218 (2.0%)
6	16 (1.3%)	52 (4.2%)	51 (4.1%)	268 (2.4%)
7	28 (2.3%)	72 (5.8%)	97 (7.8%)	465 (4.2%)
8	74 (6.0%)	146 (11.8%)	177 (14.3%)	1,143 (10.3%)
9	1,099 (88.8%)	863 (69.7%)	768 (62.0%)	8,776 (78.8%)
10	0 (0.0%)	0 (0.0%)	1 (0.1%)	2 (0.0%)
*Physical Functioning*				
0	7 (0.6%)	28 (2.3%)	38 (3.1%)	161 (1.4%)
1	19 (1.5%)	89 (7.2%)	89 (7.2%)	412 (3.7%)
2	28 (2.3%)	87 (7.0%)	95 (7.7%)	518 (4.7%)
3	61 (4.9%)	103 (8.3%)	96 (7.8%)	709 (6.4%)
4	75 (6.1%)	106 (8.6%)	119 (9.6%)	765 (6.9%)
5	79 (6.4%)	111 (9.0%)	122 (9.9%)	974 (8.7%)
6	113 (9.1%)	143 (11.6%)	119 (9.6%)	1,214 (10.9%)
7	172 (13.9%)	170 (13.7%)	151 (12.2%)	1,502 (13.5%)
8	248 (20.0%)	188 (15.2%)	171 (13.8%)	1,865 (16.8%)
9	436 (35.2%)	213 (17.2%)	238 (19.2%)	3,014 (27.1%)
***Multimorbidity***				
Cardiometabolic Conditions				
No	924 (74.6%)	547 (44.2%)	505 (40.8%)	6,432 (57.8%)
Yes	314 (25.4%)	691 (55.8%)	733 (59.2%)	4,702 (42.2%)
Neurological Conditions				
No	532 (43.0%)	237 (19.1%)	209 (16.9%)	3,108 (27.9%)
Yes	706 (57.0%)	1,001 (80.9%)	1,029 (83.1%)	8,026 (72.1%)
Musculoskeletal Conditions				
No	518 (41.8%)	265 (21.4%)	239 (19.3%)	3,332 (29.9%)
Yes	720 (58.2%)	973 (78.6%)	999 (80.7%)	7,802 (70.1%)
Respiratory Conditions				
No	1,169 (94.4%)	1,062 (85.8%)	1,045 (84.4%)	10,012 (89.9%)
Yes	69 (5.6%)	176 (14.2%)	193 (15.6%)	1,122 (10.1%)
Cancer				
No	1,074 (86.8%)	908 (73.3%)	879 (71.0%)	8,823 (79.2%)
Yes	164 (13.2%)	330 (26.7%)	359 (29.0%)	2,311 (20.8%)
***Race***				
White/Caucasian	1,056 (85.3%)	1,056 (85.3%)	1,056 (85.3%)	9,504 (85.3%)
Black/African American	154 (12.4%)	154 (12.4%)	154 (12.4%)	1,386 (12.4%)
Other	28 (2.3%)	28 (2.3%)	28 (2.3%)	252 (2.3%)
***Sex***				
Male	479 (38.7%)	479 (38.7%)	479 (38.7%)	4,311 (38.7%)
Female	759 (61.3%)	759 (61.3%)	759 (61.3%)	6,831 (61.3%)
***Education Attainment***				
less than high school	160 (12.9%)	160 (12.9%)	160 (12.9%)	1,440 (12.9%)
GED	75 (6.1%)	75 (6.1%)	75 (6.1%)	675 (6.1%)
High-school graduate	431 (34.8%)	431 (34.8%)	431 (34.8%)	3,879 (34.8%)
Some college	292 (23.6%)	292 (23.6%)	292 (23.6%)	2,628 (23.6%)
College and above	280 (22.6%)	280 (22.6%)	280 (22.6%)	2,520 (22.6%)
***Marital status***				
Unmarried	341 (27.6%)	341 (27.6%)	341 (27.6%)	3,069 (27.6%)
Married	896 (72.4%)	896 (72.4%)	896 (72.4%)	8,064 (72.4%)
***Socioeconomic Status***				
Poverty class	69 (5.6%)	130 (10.5%)	136 (11.0%)	952 (8.6%)
Lower-middle class	87 (7.0%)	83 (6.7%)	92 (7.4%)	755 (6.8%)
Middle class	133 (10.7%)	116 (9.4%)	109 (8.8%)	1,070 (9.6%)
Upper middle class	178 (14.4%)	161 (13.0%)	147 (11.9%)	1,475 (13.2%)
Wealthy	437 (35.3%)	367 (29.6%)	369 (29.8%)	3,527 (31.7%)
Super wealthy	334 (27.0%)	381 (30.8%)	385 (31.1%)	3,355 (30.1%)
Age	67.222 (5.951)	81.303 (5.959)	83.32 (5.974)	75.292 (7.910)
Body Mass Index	27.767 (5.262)	27.614 (5.663)	27.178 (5.699)	27.909 (5.586)
Drinking status	1.324 (2.211)	1.156 (2.136)	1.061 (2.097)	1.226 (2.172)
Smoking status	0.093 (0.290)	0.041 (0.199)	0.038 (0.192)	0.061 (0.239)

Table 1 omitted characteristics from 2006 to 2016. Cognitive functioning, age, drinking, body mass index, and smoking all report the means and standard deviations (in parentheses)

**Table 2:** The impact of multimorbidity on functioning trajectory

	Cognitive Functioning	Physical Functioning	Disability
*Baseline Estimates*			
Intercept	23.065***	4.461***	13.257***
Slope	0.438***	2.813***	2.672***
*Intercept Predicted by:*			
Multimorbidity (Ref no)	−0.041	−0.277***	−0.182***
Race (Ref white)	−1.599***	−0.005	−0.019
Sex (Ref male)	1.284***	−0.162***	−0.024
Education (Ref no high school)	0.924***	0.073***	−0.010
Age (Ref 60—70)	−0.055***	0.090***	0.154***
Marital Status (Ref unmarried))	−0.205	−0.020	−0.012
BMI	−0.011	−0.274***	−0.205***
Smoking (Ref no)	0.388	−0.078***	−0.101***
Drinking (Ref no)	0.017	0.018	−0.007
SES	0.581***	0.150***	0.110***
*Slope Predicted by:*			
Multimorbidity (Ref no)	−0.001	−0.052*	−0.018
Race (Ref white)	0.024	0.012	−0.053*
Sex (Ref male)	−0.033**	0.021	−0.025
Education (Ref no high school)	0.014**	0.020	0.081**
Age	−0.012***	−0.343***	−0.286***
Marital Status (Ref unmarried))	0.027	0.041	0.030
BMI	0.003*	0.020	0.005
Smoking (Ref no)	−0.039	−0.151***	−0.06*
Drinking (Ref no)	0.004	0.051*	0.036
SES	0.000	0.029	0.039
*Residual Covariance*			
Intercept and slope	−0.039*	−0.292***	−0.324
*Residual Variances*			
Intercept	5.814***	0.643***	0.806
Slope	0.032***	0.867***	0.898
Model Fit Information			
Chi-Square (*p*-value)	256.112 (0.00)	453.474 (0.00)	873.361 (0.00)
Degrees of freedom	110	110	110
RMSEA estimate	0.033	0.05	0.075
* P*-value of RMSEA < = 0.05	1	0.46	0
CFI	0.979	0.968	0.879
TLI	0.976	0.963	0.862
N	1238	1238	1238

*TLI* Tucker-Lewis Index, *CFI* Comparative Fit Index, *RSMEA* Root Mean Square Error of Approximation, *ref* Reference group

^***^ *p* < 0.01, ** *p* < 0.05, * *p* < 0.1

### Analysis

#### Growth curve modeling for the effects of multimorbidity on functioning trajectory

The first analysis utilizes growth curve modeling (GCM) to estimate both the initial levels and the rate of change in functioning trajectories among older adults in the US. GCM, which is particularly well-suited for analyzing the progression of an outcome over time, enables the estimation of individual changes while simultaneously capturing variations in these changes across the broader population [48]. By modeling both within-person and between-person differences, GCM provides a robust framework for understanding how functioning evolves and how factors such as multimorbidity influence these trajectories. This method also efficiently handles data with missingness and unequal time spacing [49].

For the GCM, we determine the coding scheme for the analysis based on the time spacing between the waves. For instance, with all variables measured at two years interval between 2004 to 2020, the time coding scheme of this study follows the sequence 0 (2004), 2, 4, 6, 8, 10, 12, 14, and 16 (2020). This coding scheme means that the first time (year 2004) is the base period of the analysis [50]. Furthermore, although age is often used to model the growth curve, Biesanz et al. [48] showed that modeling with time yields the same results, as an individual that is 60 at time 0, would be 76 at time 16.

We interpret the mean intercept as the average functioning level at the baseline period and the mean slope as the average rate of change over time. The variances and covariance of the latent growth factors (intercept and slope) capture inter-individual variability in initial functioning and change trajectories, as well as the relationship between baseline functioning and subsequent change [48].

To demonstrate well-fit GCM, we utilize the Chi-square statistics, Tucker-Lewis Index (TLI), Comparative Fit Index (CFI), and Root Mean Square Error of Approximation (RMSEA). A satisfactory model fit is established with Chi-square *p*-value > 0.05. However, with the Chi-square *p*-value mostly < 0.05 in large samples (*n* > 200), We also utilize TLI > 0.90, CFI > 0.90, and RMSEA < 0.05 to establish the model fit [48, 51, 52].

#### Mixed effects modeling for the effects of multimorbidity on older adults’ functioning

Mixed effects modeling is used to examine the association between different multimorbidity clusters (cardiometabolic, neurological, respiratory, musculoskeletal, and cancer conditions) and three healthy aging domains (cognitive functioning, physical functioning, and disability). The mixed-effects approach analyzes both fixed and random effects, addressing variations within and between individuals over time [53, 54]. This multilevel model incorporated participants' ID and wave variables, assessing the impact of multimorbidity types across models for each functioning facet. The specifications for each invividual models are detailed as follows:

1 $$\begin{aligned}{\mathit F}_{\mathit i\mathit t}\mathit\;&\mathit=\mathit\;{\mathit\pi}_{\mathit0\mathit i}\mathit\;\mathit+\mathit\;{\mathit\beta}_{\mathit1\mathit-\mathit5}\mathit M{\mathit M}_{\mathit i\mathit t}\mathit\;\mathit+\mathit\;{\mathit\beta}_{\mathit6\mathit-\mathit{10}}\mathit T\mathit V{\mathit C}_{\mathit i\mathit t}\mathit\;\\&\mathit+\mathit\;{\mathit\beta}_{\mathit{11}\mathit-\mathit{13}}\mathit T\mathit I{\mathit C}_{\mathit i}\mathit\;\mathit+\mathit\;{\mathit\beta}_{\mathit{14}\mathit-\mathit{21}}\mathit(\mathit W\mathit A\mathit V{\mathit E}_{\mathit t}\mathit\;\mathit+\mathit\;\mathit W\mathit A\mathit V{\mathit E}_{\mathit t}\mathit)\\&\mathit+\mathit\;\mathit I{\mathit D}_{\mathit i}\mathit\;\mathit+\mathit\;\mathit W\mathit A\mathit V{\mathit E}_{\mathit t}\mathit\;\mathit+\mathit\;\mathit W\mathit A\mathit V\mathit E^{\mathit2}\mathit\;_{\mathit t}\mathit\;\mathit+\mathit\;{\mathit\in}_{\mathit i\mathit t}\end{aligned}$$

Where: *i* represents the sample (1 to 1,238 individuals), *t* represents HRS waves from 2004 to 2020, and the dependent variable (*F*_*it*_) represents the functional outcomes (disability, cognitive, or physical functioning) for individual *i* at wave *t*. *TVC*_*it*_ represents the time-varying covariates (age, SES, BMI, drinking and smoking status) for individual *i* in wave *t*. *TIC*_*it*_ captures the time-invariant covariates (race, sex, and marital status) for individual *i*. *π*_*0i*​_ = *β*_0_ + µ_*0i*​_ represents the individual-level healthy aging intercept and random effects. ID is the random effect term for individual *i*, representing the deviation of individual i from the population mean. *WAVE*_*t*_ and *WAVE*^2^_*t*_ is the random effect term for time and point t, representing the deviation of time point t from the overall time trend considering that changes across time could be nonlinear. *ϵ*_*it*_ is the residual error term, representing the difference between the observed and predicted values of healthy aging. Based on available studies [55, 56], We addressed data missingness with Multivariate Imputation by Chained Equations (MICE). We also confirm that post-imputation sample statistics strongly matched the original data. We conducted all analyses using STATA 18 [54].

Additionally, to facilitate interpretation of interaction effects and group differences over time, we constructed auxiliary mixed effects models and estimated post-estimation marginal effects based on the mixed-effects models and generated predicted trajectories using marginsplot. These marginal predictions were derived from interaction terms involving time, multimorbidity clusters, and sociodemographic characteristics (e.g., sex and race). Differences between groups across time were evaluated using 95% confidence intervals around the predicted values. The graphical presentations therefore reflect model-based estimates rather than descriptive trends and are intended to aid interpretation of statistically tested effects reported in the regression tables.

#### Conditional process analysis for the mediating effects of BMI on the relationship between multimorbidity and older adults’ functioning

Because this section examines the mediating role of Body Mass Index (BMI) in promoting functioning, we employed Conditional Process Analysis, which is implemented through PROCESS Macro (version 4.2) developed for SPSS. Following the depiction of the conceptual framework in Fig. 3, two regression analyses are run for the analysis, with the first assessing path a, while the second provides the results of path b and c.

The Conditional Process Analysis used 5000 bootstrap samples to construct 95% bootstrap confidence intervals for inferential tests of indirect effects. These indirect effects show how much multimorbidity's effects are transmitted through the BMI.

Furthermore, although less than 0.1% of the data for all variables are missing (except for cognition with 22%), data missingness was handled with the SPSS Regression Based Imputation (RBI). The RBI is a method that predicts the missing values of an incomplete covariate based on multiple regression analysis of all available outcomes and explanatory variables of interest in the study model [57]. This method is advantageous because it considers the relationship between missing data and other variables while maintaining the nature and distribution of the variables, ensuring no data is lost in analysis, and helps yield unbiased estimates.

## Results

### Sample sociodemographic characteristics

Because the analytical methods used in this study required different operationalizations and data structures, Table 1 is designed for the mixed-effects regression analyses, and reports data in long format and further disaggregates multimorbidity into its constituent chronic conditions to identify the most salient disease clusters. In contrast, Table A1 (in the Supplementary File) is structured for the growth-curve modeling analyses and presents data in wide format but operationalizes multimorbidity as a binary variable distinguishing older adult with multimorbidity (two or more chronic conditions) from those without (one or none). While cross-sectional data for the base (2004) and terminal (2020) periods are used for the conditional process analysis. Using these complementary operationalizations enables the study to leverage the strengths of each analytic approach, minimize their respective limitations, and enhance the objectivity and robustness of the findings.

Across the study period, all three domains of functioning — cognitive functioning, physical functioning, and disability, declined. Mean cognitive scores dropped by 13 percentage points between baseline and terminal periods: excellent physical functioning and ability fell by 45 and 30 percentage points, respectively. When split by multimorbidity, individuals with two or more chronic conditions consistently scored lower in cognitive functioning (23.8 vs. 24.0), ability (83.7% vs. 94.6% at highest score), and physical functioning (23.7% vs. 48.4% at highest score) than those without multimorbidity.

Of the 1,238 participants in the sample, 53.5% had multimorbidity at baseline, while 46.5% did not. Chronic conditions intensified over time, with cardiometabolic conditions increasing by 130 percent, neurological by 45, musculoskeletal by 38, respiratory by 179, and cancer by 118 percent between the baseline and terminal periods.

#### Analysis of the effects of multimorbidity on the functioning trajectory of retired US older adults

Baseline estimates are fundamental for understanding the initial state and progression of older adults’ functioning. In this study, the baseline estimates, represented by intercepts and slopes indicate both the starting levels of cognitive functioning, physical functioning, and disability, as well as the rate at which they change over time. Intercepts capture the initial values at the baseline year, while slopes reflect the longitudinal trajectories observed across the nine measurement periods.

Given this conditional framework, the results in Table 2 provide insight into how the functioning trajectories evolve in the US older adult population when accounting for key factors such as multimorbidity and other demographic and socioeconomic variables. The findings highlight multimorbidity’s negative impact on functioning intercepts (the “*intercept predicted by*” portion of Table 2), especially in the physical functioning and disability domains, while its impact is not apparent in the cognitive functioning domain. However, multimorbidity does not significantly predict the rate at which older adults’ functioning decline over sixteen years (the “*slope predicted by*” portion of Table 2). These results are backed by indices such as the RMSEA, CFI and TLI, which all demonstrate model fit. The results also necessitate further investigation of the multimorbidity-functioning relationship which follows in the next subsection.

#### Analysis of the multimorbidity-stratified effects on retired older adults’ functioning

Table 3 presents the mixed effects results of the effects of multimorbidity on the functioning of retired US older adults. The results show that, apart from cognitive functioning, physical functioning and disability are both negatively influenced by multimorbidity. Besides that, the effects of multimorbidity differed by multimorbidity clusters and the functioning domain affected. For instance, the negative influence of multimorbidity showed a pronounced association with physical functioning. Also, relative to other conditions, musculoskeletal conditions are the greatest impeder of physical functioning, while respiratory conditions are the most detrimental to disability. Conversely, cancer appears to be the least instigator of functioning decline across board.

**Table 3:** Mixed-effects results of the effects of multimorbidity on retired older adults’ functioning

	Cognitive Functioning		Physical Functioning			Disability		
*Multimorbidity*								
Cardiometabolic Conditions	−0.026		−0.226	***		−0.071	**	
Neurological Conditions	−0.123		−0.350	***		−0.103	***	
Musculoskeletal Conditions	0.166		−0.742	***		−0.084	***	
Respiratory Conditions	−0.186		−0.514	***		−0.163	***	
Cancer	−0.023		−0.013			−0.036		
* Race (Ref white)*	−1.384	***	−0.297	***		−0.102	**	
* Sex (Ref male)*	0.909	***	−0.497	***		−0.024		
* Education (Ref no high school)*	0.913	***	0.190	***		0.019		
* Age*	−0.111	***	0.015	*		0.007	**	
* Marital Status (Ref unmarried))*	−0.073		0.155			0.023		
* Socioeconomic Status (Ref: Low SES)*	0.518	***	0.102	***		0.041	***	
* Body Mass Index*	0.020		−0.071	***		−0.021	***	
* Drinking (Ref no)*	0.034		0.042	***		0.014	**	
* Smoking (Ref no)*	−0.054		−0.048			−0.120	**	
*Wave (Ref: 2004)*								
2006	−0.077		−0.059			−0.024		
2008	0.027		−0.143	**		−0.028		
2010	−0.539	***	−0.326	***		−0.090	**	
2012	−0.568	***	−0.371	***		−0.108	***	
2014	−0.720	***	−0.635	***		−0.256	***	
2016	−1.003	***	−0.861	***		−0.361	***	
2018	−1.138	***	−1.244	***		−0.587	***	
2020	−1.708	***	−1.329	***		−0.861	***	
Constant	26.266	***	8.754	***		8.967	***	
Variance (Wave)	−0.861		0.585	***		0.342	***	
Variance (Wave^2)	0.08	***	0.059	***		0.003	***	
Variance (Constant)	6.502	***	1.715	***		2.942	***	
Covariance (Wave, Wave^2)	−0.077	***	−0.907	***		−0.031	***	
Covariance (Wave, Constant)	−1.081	***	−0.447	***		−0.448	***	
Covariance (Wave^2, Constant)	0.089	***	0.318	***		0.032	***	
Variance (Residuals)	8.084	***	1.168	***		1.364	***	
Number of observations	11,142		11,142		11,142			

*** *p* <.01, ** *p* <.05, * *p* <.1

**Fig. 4:**
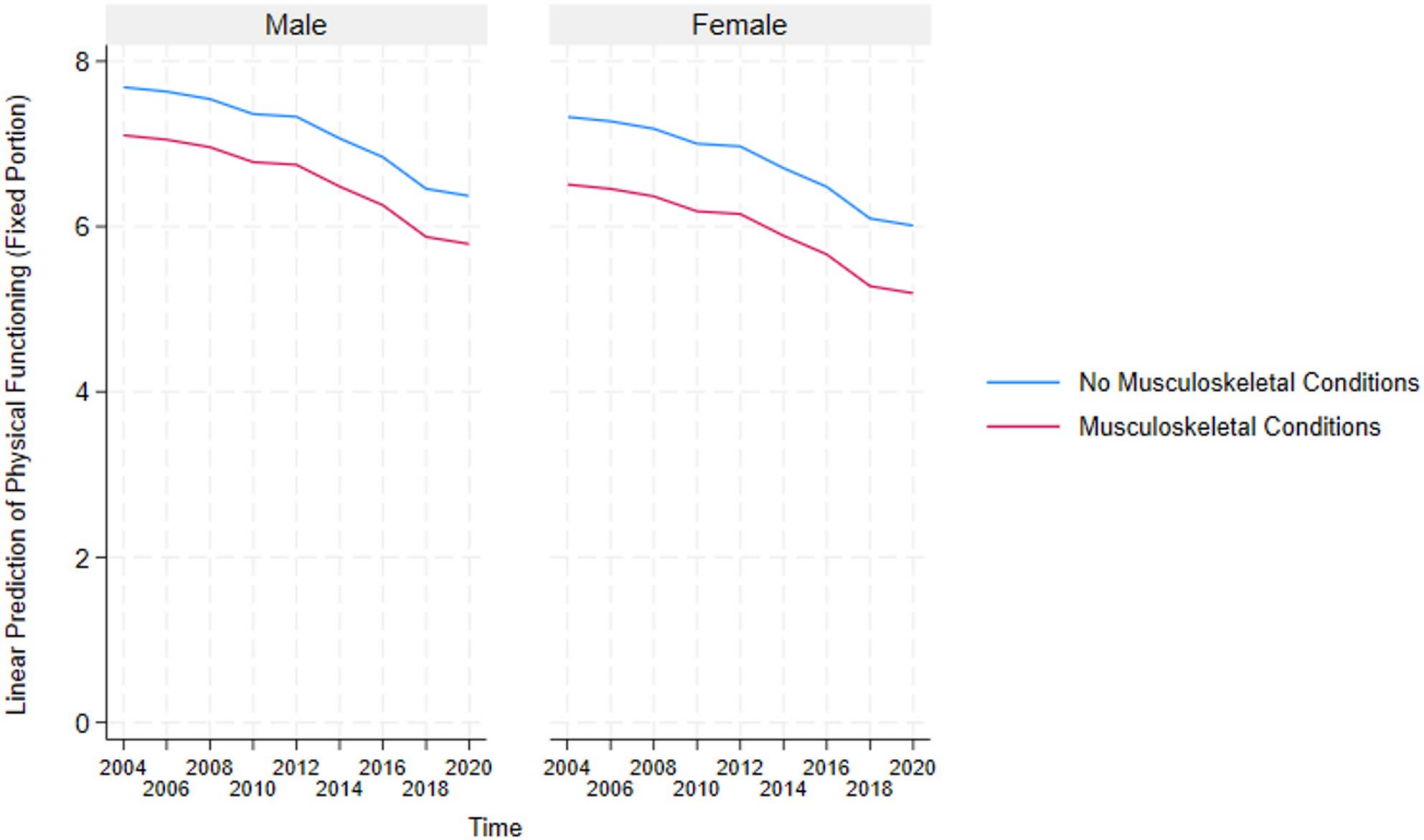
Sex differences in the effects of musculoskeletal conditions on physical functioning

**Fig. 5:**
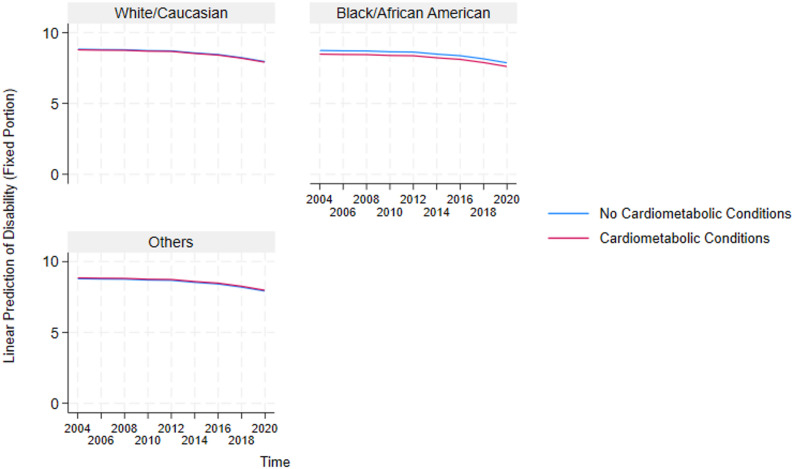
Racial differences in the effects of cardiometabolic conditions on disability

**Fig. 6:**
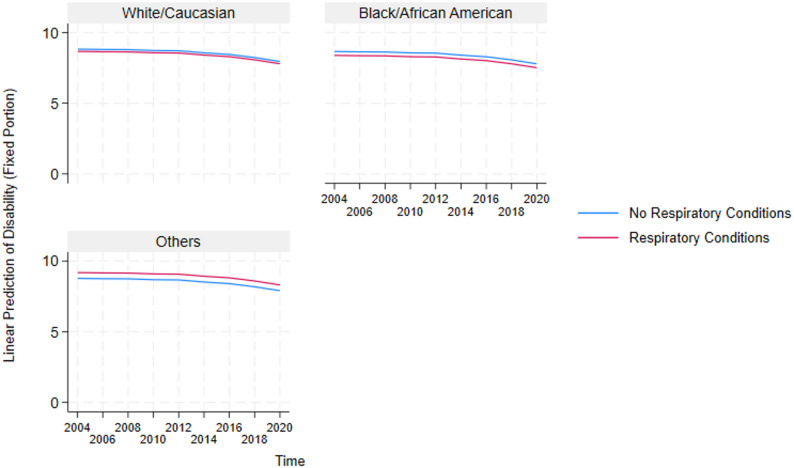
Racial differences in the effects of respiratory conditions on disability

**Fig. 7:**
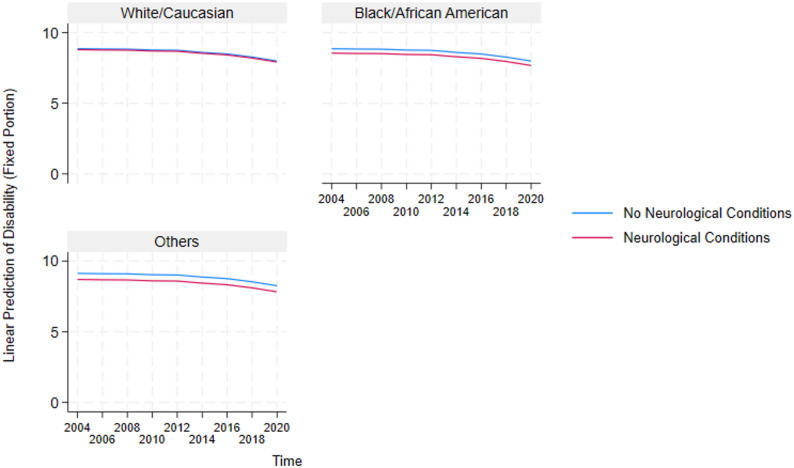
Racial differences in the effects of neurological conditions on disability

**Fig. 8:**
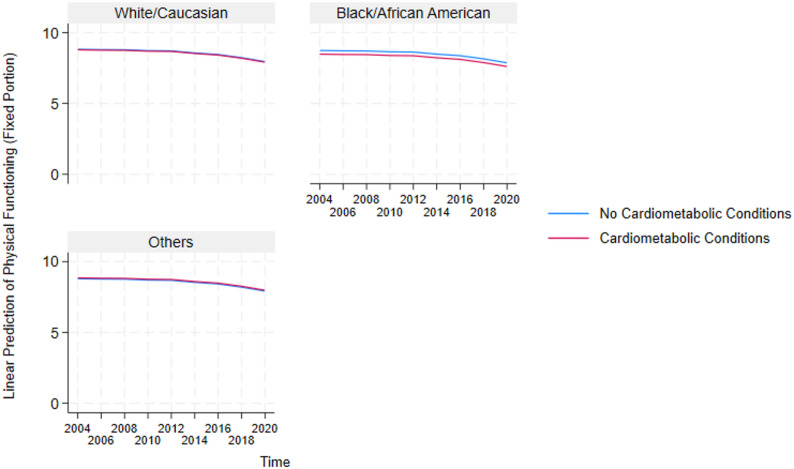
Racial differences in the effects of cardiometabolic conditions on physical functioning

**Fig. 9:**
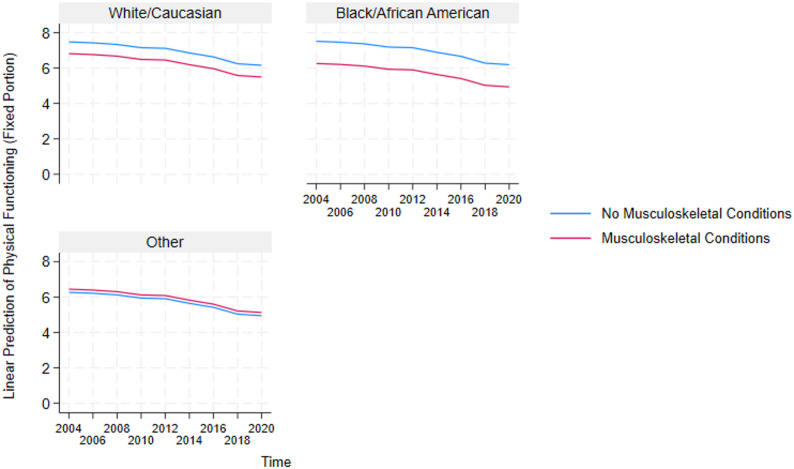
Racial differences in the effects of musculoskeletal conditions on physical functioning

These results highlight the critical detrimental effects of multimorbidity and inform the need to further understand how it impacts essential sub-groups in the US. Because multimorbidity only significantly affects the physical functioning and disability domains, we constructed auxiliary mixed-effects models (in Supplementary Table A2 and A3) for these two domains to test the negative effect of multimorbidity on the different sexes and races in the US. Additionally, post-estimation marginal predictions were computed from these models using the margins command in STATA and visualized using marginsplot. These plots (presented in Figs. 4, 5, 6, 7, 8 and 9) display model-based predicted values, facilitating interpretation of interaction effects and differences in trajectories across groups.

#### Analysis of gender-stratified effects of multimorbidity on older adults’ functioning

The results of the gender-stratified mixed-effects regression in Supplementary Table A2 show that across physical functioning and disability domains, females with multimorbidity consistently have poorer functioning. However, these effects are only significant for the musculoskeletal conditions-physical functioning association, with the marginal prediction over sixteen years plotted in Fig. 4 below. The figure shows that, relative to older adults without multimorbidity, those with multimorbidity have significantly lower physical functioning.

#### Analysis of race-stratified effects of multimorbidity on older adults’ functioning

Also, based on the race-stratified mixed-effects models in Supplementary Table A3 , Figs. 5, 6, 7, 8 and 9 depicts the statistically significant marginal predictions of the effects of multimorbidity on functioning based on the different races in the US over sixteen years. These results indicate that the US minorities tend to have poorer functioning regardless of multimorbidity cluster or functioning domain.

1) Race-Stratified Effects of Multimorbidity on Disability.

Figures 5, 6 and 7 show that Whites/Caucasians have significantly higher functioning in the disability domain than other races (with Blacks/African Americans significantly worse off) regardless of the multimorbidity status or cluster. Additionally, relative to older adults without multimorbidity, those with multimorbidity have significantly lower functioning. The figures also show that the within-group outcome differences are smaller for the White/Caucasian subgroup, revealing that the negative effects of multimorbidity are more pronounced in the minority subgroups.

2) Race-Stratified Effects of Multimorbidity on Physical functioning.

Figures 8 and 9 also indicate that Whites/Caucasians have significantly higher physical functioning than other races (with Blacks/African Americans significantly worse off) regardless of the multimorbidity status or cluster. Additionally, besides older adults without multimorbidity having better outcomes than those with multimorbidity for the White/Caucasian and Black/African American subgroups, older adults in the other race groups who have cardiometabolic and musculoskeletal conditions, tend to have higher physical functioning than those without these conditions. The figures also show that the within-group outcome differences are smaller for the White/Caucasian subgroup, revealing that the negative effects of multimorbidity are more pronounced in the minority subgroups.

#### Analysis of the mediating effects of body mass index on the relationship between multimorbidity and older adults’ functioning

This section employed Hayes’ Conditional Process Analysis (PROCESS Model 4) to investigate the mediating role of Body Mass Index (BMI) in the relationship between multimorbidity and three functioning domains — cognitive functioning, physical functioning, and disability—among older adults in the United States. By applying PROCESS Model 4, the analysis estimated both the direct effects of multimorbidity on functioning and the indirect effects transmitted through BMI, thereby allowing for a rigorous assessment of mediation across time. Because this method does not permit longitudinal analysis, we investigated this relationship for the base and terminal periods to provide adequate perspective of the research objective. This approach provides a nuanced understanding of the pathways by which multiple chronic conditions shape late-life health outcomes and informs the discussion of how BMI contributes to, or buffers these effects.

#### The mediating effects of body mass index on the relationship between multimorbidity and the cognitive functioning domain

**Fig. 10:**
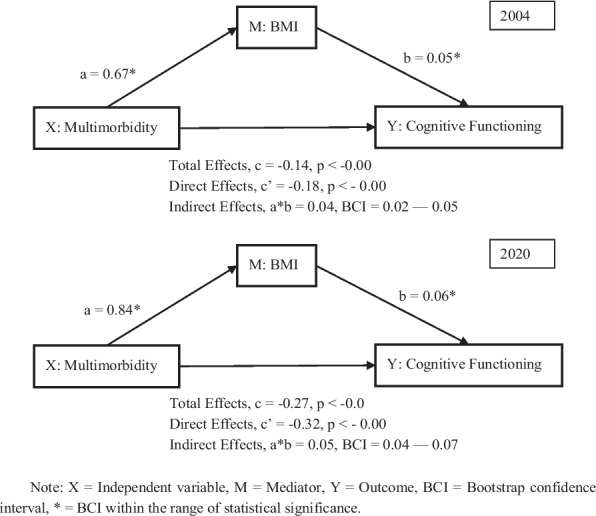
The mediating effects of BMI on cognitive functioning in 2004 and 2020

Supplementary Table A4 and Fig. 10 report the mediating effects of BMI on the cognitive functioning domain Across both 2004 and 2020 in the cognitive functioning domain, multimorbidity was significantly and positively associated with BMI (a = 0.67 in 2004; a = 0.84 in 2020), indicating that older adults with multimorbidity tend to have higher BMI. Similarly, BMI was positively associated with cognitive functioning (b = 0.05 in 2004; b = 0.06 in 2020), suggesting that older adults with higher BMI potentially have better cognition. The total effect of multimorbidity on cognitive functioning was negative and significant (c = –0.14 in 2004; c = –0.27 in 2020), showing the adverse effects of multimorbidity on older adults’ cognition. Moreover, the direct effect remained negative after controlling for BMI (c’ = –0.18 in 2004; c’ = –0.32 in 2020), while the indirect effect of multimorbidity through BMI was statistically significant in both years (a*b = 0.04, 95% CI = 0.02–0.05 in 2004; ab = 0.05, 95% CI = 0.04–0.07 in 2020), indicating a small but consistent mediating role of BMI. Control variables behaved as expected: education and socioeconomic status (SES) were positively associated with cognitive functioning, while age and smoking were negatively associated.

#### The mediating effects of body mass index on the relationship between multimorbidity and the physical functioning domain

**Fig. 11:**
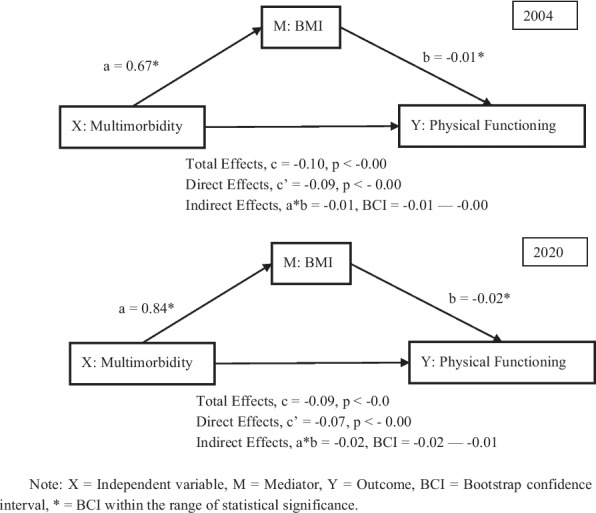
The mediating effects of BMI on physical functioning in 2004 and 2020

Supplementary Table A5 and Fig. 11 account for the mediating effects of BMI on the physical functioning domain. Across both 2004 and 2020, multimorbidity was significantly and positively associated with BMI (a = 0.67 in 2004; a = 0.84 in 2020), indicating that older adults with multimorbidity tend to have higher BMI. In turn, BMI was negatively associated with physical functioning (b = –0.01 in 2004; b = –0.02 in 2020), suggesting that older adults with higher BMI had poorer physical functioning. The total effect of multimorbidity on physical functioning was negative and significant (c = –0.10 in 2004; c = –0.09 in 2020), reflecting the adverse impact of multimorbidity on older adults’ physical functioning. Moreover, the direct effect remained negative after controlling for BMI (c’ = –0.09 in 2004; c’ = –0.07 in 2020), while the indirect effect of multimorbidity through BMI was also statistically significant in both years (a*b = –0.01, 95% CI = –0.01 to –0.00 in 2004; ab = –0.02, 95% CI = –0.02 to –0.01 in 2020), indicating a small but consistent mediating role of BMI. Control variables also behaved as expected: age and smoking were negatively associated with physical functioning, while drinking and socioeconomic status (SES) were positively associated.

#### The mediating effects of body mass index on the relationship between multimorbidity and the disability domain

**Fig. 12:**
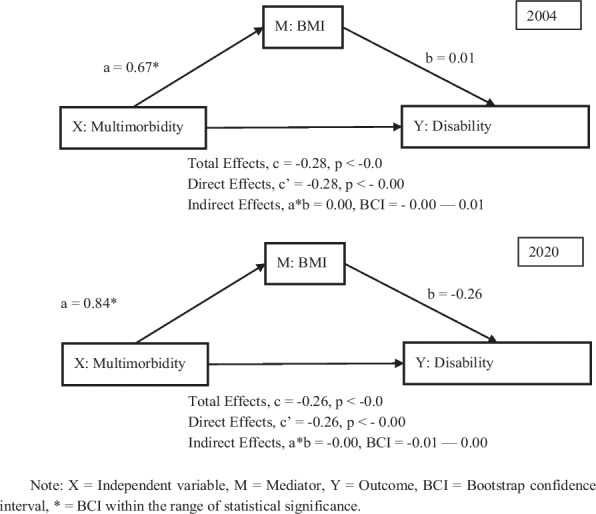
The mediating effects of BMI on disability in 2004 and 2020

Furthermore, Supplementary Table A6 and Fig. 12 report the mediating effects of BMI on the disability domain. Across 2004 and 2020, multimorbidity was significantly and positively associated with BMI (a = 0.67 in 2004; a = 0.84 in 2020), again indicating that older adults with multimorbidity tend to have higher BMI. However, BMI showed almost no association with disability (b = 0.01 in 2004; b = –0.26 in 2020 with confidence intervals overlapping zero), suggesting that BMI did not substantially influence disability levels. The total effect of multimorbidity on disability was negative and significant (c = –0.28 in 2004; c = –0.26 in 2020), reflecting the adverse effect of multimorbidity on older adults’ disability outcomes. Moreover, the direct effect remained essentially unchanged after controlling for BMI (c’ = –0.28 in 2004; c’ = –0.26 in 2020), while the indirect effect of multimorbidity through BMI was negligible and statistically non-significant in both years (a*b = 0.00, 95% CI crosses zero in 2004; ab = –0.00, 95% CI crosses zero in 2020), indicating no mediating role of BMI in the disability domain. Control variables behaved as expected: marital status, education, and SES were positively associated with lower disability, while age and smoking were negatively associated.

Taken together, the figures and tables indicate that BMI consistently mediates part of the relationship between multimorbidity and both cognitive and physical functioning but not disability. The mediation effects became slightly stronger between 2004 and 2020 for cognitive and physical functioning, suggesting BMI’s role may have grown as the population aged or BMI distributions changed over time. R-squared values were highest for cognitive functioning models (0.33 in 2004; 0.26 in 2020), moderate for disability (0.17 and 0.12), and lowest for physical functioning (0.05 and 0.04), reflecting domain-specific variance explained.

## Discussion

This study examined how multimorbidity, measured through disease clusters and binary indicators, shapes retired older adults’ functioning in the United States using three analytical strategies: growth curve modeling, mixed-effects regression, and conditional process analysis. Across these approaches, multimorbidity is consistently a key determinant of functioning. Growth curve models showed that multimorbidity is associated with lowers baseline levels of functioning across physical functioning and disability domains but does not significantly affect its rate of decline (across all domains) over a sixteen-year period. Mixed-effects models further confirmed strong negative associations between multimorbidity, physical functioning and disability, alongside notable variation across disease clusters. Finally, mediation analyses demonstrated that Body Mass Index (BMI) partially explains the impact of multimorbidity on physical and cognitive functioning but not disability. Taken together, these findings highlight both the early-life imprint and the domain-specific pathways through which multimorbidity undermines functioning. Importantly, these patterns are specially observed among older adults that retired involuntarily, a life-course stage characterized by changes in daily routines, health behaviors, and physical activity levels following withdrawal from the labor force, which may shape the ways chronic disease burden translates into functional outcomes [58, 59].

The growth modeling results reveal that the principal disadvantage associated with multimorbidity manifests at baseline rather than through accelerated deterioration. Specifically, besides cognitive functioning, multimorbidity is associated with lower baseline of physical functioning and disability. Multimorbidity is more likely to immediately impact and predict baseline physical functioning and disability because many chronic conditions directly impair musculoskeletal, cardiovascular, and metabolic systems that affect mobility and the ability to perform ADL/IADL, producing observable baseline functional limitations [7, 9]. Contrastingly, evidence linking multimorbidity to cognitive functioning is less consistent, as the effects of multimorbidity on baseline cognition are less likely to be immediate (or non-significant) even across several chronic conditions [10]. Additionally, multimorbidity does not significantly affect the slope of all functioning domains across the sixteen-year follow-up. This pattern aligns with cumulative disadvantage theory, which posits that inequalities become embedded early in life and shape later-life trajectories [60]. Thus, individuals who enter older adulthood with multimorbidity appear to carry a substantial functional deficit that persists rather than widens across time. In the context of retirement, this baseline disadvantage may be particularly consequential because the transition out of employment can coincide with reductions in structured physical activity and daily mobility, potentially amplifying the functional consequences of chronic disease [9, 58].

Building on the growth curve findings, the mixed-effects models provide additional clarity by identifying domain-specific and disease-cluster–specific associations. These models show that multimorbidity is negatively associated with physical functioning and disability, but there is no statistically significant effect on cognitive functioning. Within this broader pattern, the results reveal variation across disease clusters. For example, musculoskeletal conditions were associated with comparatively larger reductions in physical functioning, consistent with previous evidence linking arthritis, osteoporosis, and chronic pain syndromes to mobility limitations, loss of independence, and diminished quality of life [9, 61, 62]. Likewise, respiratory conditions were associated with notably higher levels of disability, echoing research showing that chronic obstructive pulmonary disease (COPD) and asthma accelerate declines in ADL and [63]. These findings are particularly relevant for retired populations, as the absence of occupational physical demands may shift the determinants of functioning toward health-related limitations and disease management rather than workplace-related activity levels [58]. These patterns suggest that interventions aimed at preserving muscle strength, joint health, and pulmonary capacity, such as tailored physical activity programs, early rehabilitation, and aggressive chronic disease management, could substantially mitigate declines in functioning [10].

The results stratified by sex and race further contextualize these effects and highlight persistent demographic disparities. Consistent with gendered patterns in chronic disease exposure, caregiving burden, and healthcare utilization, males display higher levels of physical functioning and lower disability than females regardless of multimorbidity status or cluster [39]. Although the differences are not statistically significant for most conditions, there is urgent need for sex-specific intervention strategies for older females before the potential health inequity widens significantly. These disparities may become more visible after retirement, when differences in lifetime occupational exposures, income security, and access to healthcare accumulate and shape health trajectories [58]. Similarly, race-stratified analyses reveal that White/Caucasian older adults exhibit significantly higher levels of functioning across physical functioning and disability domains compared to Black/African American and other racial groups. These findings mirror structural inequities in healthcare access, socioeconomic position, and cumulative exposure to adverse social determinants of health [38]. Moreover, within-group differences are smaller for Whites, suggesting that the negative effects of multimorbidity are amplified among minority populations. These disparities strengthen the case for culturally tailored and equity-focused interventions that address both disease management and the broader social context in which older adults age.

To deepen the understanding of mechanisms underlying these associations, the conditional process analysis sheds light on the mediating role of BMI in the relationship between multimorbidity and functioning. The analysis reveals that BMI consistently mediates part of the relationship between multimorbidity, cognitive and physical functioning. For cognition, multimorbidity is positively associated with BMI, and BMI in turn shows a small but significant positive association with cognitive functioning. Although the total effect of multimorbidity on cognition remains negative, BMI mediates a modest portion of this relationship. This pattern echoes research on the complex late-life relationship between adiposity and cognitive outcomes, including the “obesity paradox,” which describes situations in which higher BMI appears protective due to survival bias or reflects better nutritional status among frail older adults [33]. Although this phenomenon is usually observed in physical outcomes, some studies suggest that higher BMI in late life may be associated with slower decline in cognitive functioning [64], though this relationship is complex and requires cautious interpretation. In the post-retirement context, BMI may also reflect changes in lifestyle, diet, and activity patterns that often accompany the transition out of the workforce [65].

A similar but more pronounced pattern emerges for physical functioning. Multimorbidity increases BMI, and higher BMI is consistently associated with poorer physical functioning. Significant indirect effects indicate that BMI partially mediates the multimorbidity–physical functioning pathway. This is consistent with evidence showing that higher BMI exacerbates musculoskeletal strain, inflammation, and pain, which in turn accelerate functional decline [41, 66, 67]. BMI also increases joint load and reduces activity tolerance, thereby quickening losses in mobility and muscle strength [68]. The slightly stronger mediation effects observed across the two periods point to the growing influence of BMI as a risk factor in an aging and increasingly overweight population. This finding supports cumulative disadvantage theory, wherein health risks compound over time to shape late-life outcomes. Among retired adults, such risks may accumulate more rapidly when declines in occupational physical activity are not offset by recreational or preventive health behaviors [10, 58].

Contrastingly, although multimorbidity is associated with BMI, BMI shows negligible associations with disability, and indirect effects are non-significant. This suggests that disability, measured through ADL and IADL limitations, is more directly influenced by disease severity, neurological impairment, pain burden, and entrenched functional losses than by weight status alone [69–71]. This divergence between physical functioning and disability highlights an important clinical distinction: physical functioning reflects current mobility and strength, which BMI can plausibly affect, whereas disability represents more persistent and often irreversible limitations. As such, weight management may improve physical functioning but is unlikely to reverse established disability without additional rehabilitative or compensatory interventions, such as disease-specific rehabilitation, assistive devices, or environmental modifications. These distinctions are especially relevant for retirees, whose independence and quality of life often depend heavily on the ability to maintain functional autonomy outside the structure of employment [65].

Overall, these findings carry important implications for policy and practice. Identifying BMI as an actionable mediator in the multimorbidity–functioning relationship for cognitive and physical functioning provides a rationale for integrating weight management into multimorbidity care plans for retired older adults. However, interventions focused solely on BMI are unlikely to prevent or reverse disability once it emerges; additional rehabilitative and compensatory strategies are needed to preserve independence. Because BMI’s mediating role appears stronger in the terminal period compared to baseline, addressing weight-related risk factors in midlife or early old age may provide meaningful benefits for functional health. Moreover, health systems should consider stratifying older adults not only by multimorbidity burden but also by BMI and domain-specific functioning deficits to better target preventive and therapeutic resources. Given that the study population consists of retired individuals, these strategies are particularly important for sustaining independence and well-being in the post-retirement phase of life, when maintaining functioning becomes central to healthy aging and continued community participation.

### Study limitations

Firstly, a key limitation is that BMI was modeled as a time-specific mediator without incorporating curvilinear specifications or time-varying change, limiting our ability to capture nonlinear effects and dynamic weight trajectories. However, by estimating mediation effects at both the baseline and terminal periods, the analysis still captures potential differences between early and later stages of the observation window, providing insight into whether BMI’s mediating role strengthens or weakens over time even without explicitly modeling continuous BMI trajectories.

Secondly, disease clusters were constructed using condition-level variables available in the HRS, which does not differentiate clinical subtypes within broad categories such as musculoskeletal, cancer, or respiratory conditions. Although these categories represent clinically heterogeneous groupings, the absence of finer diagnostic detail limits our ability to assess within-cluster variability and may attenuate distinctions between clusters.

## Conclusions

This study demonstrates that multimorbidity exerts a powerful and enduring influence on older adults’ functioning, shaping their trajectories long before late-life decline becomes apparent. By integrating growth curve, mixed-effects, and mediation analyses, the findings reveal that multimorbidity primarily undermines functioning through substantial baseline disadvantages rather than accelerated deterioration over time. Distinct disease clusters, particularly musculoskeletal and respiratory conditions, as critical drivers of functional loss, while persistent sex and racial disparities underscore the role of structural and social inequities in shaping late-life outcomes. BMI further operates as a domain-specific mechanism, partially mediating the effects of multimorbidity on cognitive and physical functioning but not disability, highlighting both the modifiable and entrenched components of functional decline.

Together, these results advance theoretical and empirical understanding of functioning in older adulthood by clarifying when multimorbidity matters most, which conditions exert the greatest burden, and how intermediary pathways differ across functional domains. They point to the necessity of early and life-course–oriented prevention, targeted and disease-specific management strategies, and equity-focused policies that address the intersecting influences of biology, behavior, and structural context. Interventions aimed at weight management, musculoskeletal and respiratory rehabilitation, and social and environmental supports hold promise for mitigating the functional consequences of multimorbidity. As populations age and multimorbidity become increasingly prevalent, these insights offer critical guidance for designing more effective, equitable, and sustainable approaches to preserving functioning and independence in later life.

## Supplementary Information

Supplementary Material 1.

## Data Availability

Publicly available datasets were used in this study. These can be found in [Health and Retirement Study Website] at [https://hrsdata.isr.umich.edu/data-products/rand-hrs-longitudinal-file-2022] Further enquiries regarding the analysis codes can be directed to the corresponding author.

## Abbreviations

HRS: Health and Retirement Study
ADL: Activities of Daily Living
IADL: Instrumental Activities of Daily Living
BMI: Body Mass Index
GCM: Growth curve models

## Footnotes

1 Cumulative advantage/disadvantage theory posits that initial social, economic, or health-related inequalities systematically widen over the life course as advantages compound into greater resources and opportunities while disadvantages accumulate into escalating risks and constraints.

2 The eight chronic conditions are as follows: “1) high blood pressure or hypertension; 2) diabetes or high blood sugar; 3) cancer or a malignant tumor of any kind except skin cancer; 4) chronic lung disease except asthma such as chronic bronchitis or emphysema; 5) heart attack, coronary heart disease, angina, congestive heart failure, or other heart problems; 6) stroke or transient ischemic attack (TIA); 7) emotional, nervous, or psychiatric problems; and 8) arthritis or rheumatism.”
