# Supplementary material for: Multimorbidity and functioning disparities in retired older adults: longitudinal trajectories, disease clusters, and mediating mechanisms
Source: BMC Geriatr. 2026 Apr 6;26:698. doi: 10.1186/s12877-026-07388-9 (PMC13191954; doi:10.1186/s12877-026-07388-9)
Supplement: Supplementary file 1 — Supplementary Material 1. [file 12877_2026_7388_MOESM1_ESM.docx]

Table A1: Sample Sociodemographic Characteristics based on wide data format

|  | Multimorbidity | | |
| --- | --- | --- | --- |
|  | No Multimorbidity | Multimorbidity | Total |
| N | 2,739 (31.7%) | 5,915 (68.3%) | 8,654 (100.0%) |
| Cognition | 21.856 (5.046) | 20.852 (5.202) | 21.170 (5.174) |
| Disability |  |  |  |
| 0.Difficulty in all tasks | 0 (0.0%) | 29 (0.5%) | 29 (0.3%) |
| 1 | 12 (0.4%) | 61 (1.0%) | 73 (0.8%) |
| 2 | 13 (0.5%) | 62 (1.0%) | 75 (0.9%) |
| 3 | 13 (0.5%) | 104 (1.8%) | 117 (1.4%) |
| 4 | 19 (0.7%) | 147 (2.5%) | 166 (1.9%) |
| 5 | 39 (1.4%) | 276 (4.7%) | 315 (3.6%) |
| 6 | 47 (1.7%) | 306 (5.2%) | 353 (4.1%) |
| 7 | 71 (2.6%) | 403 (6.8%) | 474 (5.5%) |
| 8 | 174 (6.4%) | 807 (13.6%) | 981 (11.3%) |
| 9 | 2,351 (85.8%) | 3,717 (62.9%) | 6,068 (70.1%) |
| 10.Ability with all tasks | 0 (0.0%) | 1 (0.0%) | 1 (0.0%) |
| Physical Functioning |  |  |  |
| 0.Poor physical Functioning | 1 (0.0%) | 1 (0.0%) | 2 (0.0%) |
| 1 | 6 (0.2%) | 39 (0.7%) | 45 (0.5%) |
| 2 | 36 (1.3%) | 185 (3.1%) | 221 (2.6%) |
| 3 | 123 (4.5%) | 650 (11.0%) | 773 (8.9%) |
| 4 | 305 (11.1%) | 1,106 (18.7%) | 1,411 (16.3%) |
| 5 | 1,385 (50.6%) | 1,910 (32.3%) | 3,295 (38.1%) |
| 6 | 543 (19.8%) | 1,232 (20.9%) | 1,775 (20.5%) |
| 7 | 267 (9.8%) | 611 (10.3%) | 878 (10.2%) |
| 8 | 63 (2.3%) | 151 (2.6%) | 214 (2.5%) |
| 9.Excellent Physical Functioning | 7 (0.3%) | 22 (0.4%) | 29 (0.3%) |
| Marital Status |  |  |  |
| 0.Unmarried | 943 (34.4%) | 2,504 (42.4%) | 3,447 (39.9%) |
| 1.Married | 1,796 (65.6%) | 3,405 (57.6%) | 5,201 (60.1%) |
| Drinking Status |  |  |  |
| 0.Doesnt drink | 1,725 (63.1%) | 4,384 (74.2%) | 6,109 (70.7%) |
| 1.One day per week | 248 (9.1%) | 482 (8.2%) | 730 (8.4%) |
| 2.Twice a week | 172 (6.3%) | 213 (3.6%) | 385 (4.5%) |
| 3.Thrice a week | 141 (5.2%) | 200 (3.4%) | 341 (3.9%) |
| 4.Four days a week | 84 (3.1%) | 88 (1.5%) | 172 (2.0%) |
| 5.Five days a week | 66 (2.4%) | 88 (1.5%) | 154 (1.8%) |
| 6.Six days a week | 37 (1.4%) | 51 (0.9%) | 88 (1.0%) |
| 7.Everyday | 261 (9.5%) | 401 (6.8%) | 662 (7.7%) |
| Smoking Status |  |  |  |
| 0.No Smoking | 2,357 (86.9%) | 5,188 (88.5%) | 7,545 (88.0%) |
| 1.Smokes | 356 (13.1%) | 674 (11.5%) | 1,030 (12.0%) |
| Age | 71.604 (9.025) | 73.619 (9.305) | 72.981 (9.265) |
| Total Wealth | 912,134.528 (1,496,881.170) | 519,526.644 (1,052,544.221) | 589,880.632 (1,154,669.644) |
| Body Mass Index | 25.869 (4.685) | 27.534 (5.916) | 27.006 (5.609) |

Cognitive functioning, age, body mass index, and wealth all report the means and standard deviations (in parentheses)

| Table A2: Sex Differences in the Effects of Multimorbidity | | | | |
| --- | --- | --- | --- | --- |
|  | Physical Functioning | | Disability | |
| *Sex (Ref male)* | -0.261 | * | 0.070 |  |
| *Multimorbidity* |  |  |  |  |
| Cardiometabolic Conditions | -0.171 | * | -0.077 | * |
| *Cardiometabolic Conditions*Female* | -0.053 |  | 0.017 |  |
| Neurological Conditions | -0.268 | *** | -0.046 |  |
| *Neurological Conditions*Female* | -0.107 |  | -0.076 |  |
| Musculoskeletal Conditions | -0.582 | *** | -0.030 |  |
| *Musculoskeletal Conditions*Female* | -0.236 | * | -0.082 |  |
| Respiratory Conditions | -0.513 | *** | -0.056 |  |
| *Respiratory Conditions*Female* | 0.002 |  | -0.158 |  |
| Cancer | -0.008 |  | -0.039 |  |
| *Cancer*Female* | 0.004 |  | 0.010 |  |
| Race (Ref white) | -0.293 | *** | -0.092 | ** |
| Education (Ref no high school) | 0.193 | *** | 0.022 |  |
| Age | 0.012 |  | 0.007 | ** |
| Marital Status (Ref unmarried)) | 0.145 |  | 0.019 |  |
| Socioeconomic Status (Ref: Low SES) | 0.106 | *** | 0.040 | *** |
| Body Mass Index | -0.074 | *** | -0.022 | *** |
| Drinking (Ref no) | 0.042 | *** | 0.013 | ** |
| Smoking (Ref no) | -0.024 |  | -0.115 | ** |
| Wave (Ref: 2004) |  |  |  |  |
| 2006 | -0.053 |  | -0.023 |  |
| 2008 | -0.143 | ** | -0.035 |  |
| 2010 | -0.324 | *** | -0.095 | ** |
| 2012 | -0.356 | *** | -0.112 | *** |
| 2014 | -0.620 | *** | -0.259 | *** |
| 2016 | -0.846 | *** | -0.373 | *** |
| 2018 | -1.229 | *** | -0.594 | *** |
| 2020 | -1.316 | *** | -0.874 | *** |
| Constant | 8.325 | *** | 8.901 | *** |
| Variance (Wave) | 0.334 |  | 0.126 |  |
| Variance (Wave^2) | 0.003 |  | 0.002 |  |
| Variance (Constant) | 2.925 |  | 0.354 |  |
| Covariance (Wave, Wave^2) | -0.030 | *** | -0.014 | *** |
| Covariance (Wave, Constant) | -0.439 | *** | -0.133 | *** |
| Covariance (Wave^2, Constant) | 0.031 | *** | 0.015 | *** |
| Variance (Residuals) | 1.360 |  | 0.459 |  |
| Number of observations | 10940 |  | 10939 |  |
| *** p<.01, ** p<.05, * p<.1 my note | | | | |

| Table A3: Racial Differences in the Effects of Multimorbidity | | | | |
| --- | --- | --- | --- | --- |
|  | Physical Functioning | | Disability | |
| Race (Ref white) |  |  |  |  |
| Black/African American | 0.009 |  | 0.230 | * |
| Others | -0.905 |  | 0.236 |  |
| *Multimorbidity* |  |  |  |  |
| Cardiometabolic Conditions | -0.194 | *** | -0.041 |  |
| Cardiometabolic Conditions* Black/African American | -0.190 |  | -0.219 | ** |
| Cardiometabolic Conditions*Others | 0.949 | ** | 0.102 |  |
| Neurological Conditions | -0.348 | *** | -0.070 | ** |
| Neurological Conditions* Black/African American | 0.280 |  | -0.241 | ** |
| Neurological Conditions*Others | -0.890 |  | -0.360 |  |
| Musculoskeletal Conditions | -0.662 | *** | -0.062 | * |
| Musculoskeletal Conditions* Black/African American | -0.591 | *** | -0.128 |  |
| Musculoskeletal Conditions*Others | 0.842 | * | -0.123 |  |
| Respiratory Conditions | -0.519 | *** | -0.160 | *** |
| Respiratory Conditions* Black/African American | -0.159 |  | -0.114 |  |
| Respiratory Conditions*Others | 0.934 |  | 0.568 | * |
| Cancer | 0.049 |  | -0.014 |  |
| Cancer* Black/African American | -0.383 |  | -0.199 | * |
| Cancer*Others | -0.754 |  | 0.071 |  |
| *Sex (Ref male)* | -0.499 | *** | -0.018 |  |
| Education (Ref no high school) | 0.199 | *** | 0.023 |  |
| Age | 0.013 | * | 0.006 | ** |
| Marital Status (Ref unmarried)) | 0.150 |  | 0.016 |  |
| Socioeconomic Status (Ref: Low SES) | 0.106 | *** | 0.037 | *** |
| Body Mass Index | -0.074 | *** | -0.022 | *** |
| Drinking (Ref no) | 0.040 | *** | 0.012 | ** |
| Smoking (Ref no) | -0.018 |  | -0.118 | ** |
| Wave (Ref: 2004) |  |  |  |  |
| 2006 | -0.054 |  | -0.023 |  |
| 2008 | -0.145 | ** | -0.033 |  |
| 2010 | -0.327 | *** | -0.095 | ** |
| 2012 | -0.361 | *** | -0.112 | *** |
| 2014 | -0.623 | *** | -0.258 | *** |
| 2016 | -0.849 | *** | -0.372 | *** |
| 2018 | -1.234 | *** | -0.594 | *** |
| 2020 | -1.321 | *** | -0.873 | *** |
| Constant | 8.568 | *** | 8.870 | *** |
| Variance (Wave) | 0.334 |  | 0.124 |  |
| Variance (Wave^2) | 0.003 |  | 0.002 |  |
| Variance (Constant) | 2.900 |  | 0.348 |  |
| Covariance (Wave, Wave^2) | -0.030 | *** | -0.014 | *** |
| Covariance (Wave, Constant) | -0.442 | *** | -0.132 | *** |
| Covariance (Wave^2, Constant) | 0.032 | *** | 0.015 | *** |
| Variance (Residuals) | 1.360 |  | 0.459 |  |
| Number of observations | 10940 |  | 10939 |  |
| *** p<.01, ** p<.05, * p<.1 my note | | | | |

Table A4 The mediating effects of BMI on the cognitive functioning domain

|  | Cognitive Functioning Domain | | |  |
| --- | --- | --- | --- | --- |
|  | 2004 | Bootstrap CI | 2020 | Bootstrap CI |
| Constant | 28.04 | 26.73 — 29.31 | 25.68 | 24.25 — 27.07 |
| Multimorbidity | -0.18 | -0.25 — -0.12 | -0.32 | -0.40 — -0.25 |
| BMI *(Ref: No Conditions)* | 0.05 | 0.04 — 0.07 | 0.06 | 0.05 — 0.08 |
| Marital Status *(Ref: Unmarried)* | -0.16 | -0.37 — 0.06 | 0.89 | 0.66 — 1.12 |
| Sex *(Ref: Male)* | 1.01 | 0.83 — 1.21 | 1.15 | 0.93 — 1.38 |
| Race *(Ref: White)* | -1.78 | -20 — -1.57 | -1.15 | -1.33 — -0.97 |
| *Education Attainment (Ref: < High School)* | 0.93 | 0.86 — 1.01 | 1.26 | 1.17 — 1.34 |
| Age | -0.16 | -0.17 — -0.15 | -0.14 | -0.15 — -0.12 |
| Smoking *(Ref: No smoking)* | -0.09 | -0.39 — 0.20 | -0.68 | -1.05 — -0.31 |
| Drinking *(Ref: No drinking)* | 0.13 | 0.09 — 0.18 | 0.13 | 0.08 — 0.18 |
| SES | 0.63 | 0.56 — 0.70 | 0.00 | 0.00 — 0.00 |
| R-square | 0.33 |  | 0.26 |  |
| F | 475.59 |  | 301.24 |  |
| N | 8654 |  | 7941 |  |

Note: 95% bootstrap confidence intervals are reported

Table A5 The mediating effects of BMI on the physical functioning domain

|  | Physical Functioning Domain | | |  |
| --- | --- | --- | --- | --- |
|  | 2004 | Bootstrap CI | 2020 | Bootstrap CI |
| Constant | 6.84 | 6.45 — 7.23 | 7.12 | 6.73 — 7.51 |
| Multimorbidity | -0.09 | -0.11 — -0.07 | -0.07 | -0.09 — -0.05 |
| BMI *(Ref: No Conditions)* | -0.01 | -0.02 — -0.01 | -0.02 | -0.03 — -0.02 |
| Marital Status *(Ref: Unmarried)* | 0.10 | 0.04 — 0.17 | 0.09 | 0.03 — 0.15 |
| Sex *(Ref: Male)* | 0.08 | 0.03 — 0.14 | -0.03 | -0.09 — 0.03 |
| Race *(Ref: White)* | -0.01 | -0.07 — 0.06 | 0.07 | 0.03 — 0.12 |
| *Education Attainment (Ref: < High School)* | -0.03 | -0.06 — -0.01 | -0.01 | -0.03 — 0.02 |
| Age | -0.02 | -0.02 — -0.02 | -0.02 | -0.02 — -0.02 |
| Smoking *(Ref: No smoking)* | -0.22 | -0.31 — -0.13 | -0.18 | -0.27 — -0.08 |
| Drinking *(Ref: No drinking)* | 0.01 | 0.00 — 0.02 | 0.02 | 0.01 — 0.03 |
| SES | 0.07 | 0.05 — 0.09 | 0.00 | 0.00 — 0.00 |
| R-square | 0.05 |  | 0.04 |  |
| F | 51.35 |  | 33.42 |  |
| N | 8654 |  | 7941.00 |  |

Note: 95% bootstrap confidence intervals are reported

Table A6 The mediating effects of BMI on the disability domain

|  | Disability Domain | | |  |
| --- | --- | --- | --- | --- |
|  | 2004 | Bootstrap CI | 2020 | Bootstrap CI |
| Constant | 9.84 | 9.31 — 10.38 | 9.84 | 9.31 — 10.38 |
| Multimorbidity | -0.26 | -0.28 — -0.23 | -0.26 | -0.28 — -0.23 |
| BMI *(Ref: No Conditions)* | -0.01 | -0.01 — 0.00 | -0.01 | -0.01 — 0.00 |
| Marital Status *(Ref: Unmarried)* | 0.23 | 0.15 — 0.31 | 0.23 | 0.15 — 0.31 |
| Sex *(Ref: Male)* | -0.05 | -0.12 — 0.02 | -0.05 | -0.12 — 0.02 |
| Race *(Ref: White)* | -0.08 | -0.14 — -0.02 | -0.08 | -0.14 — -0.02 |
| *Education Attainment (Ref: < High School)* | 0.12 | 0.08 — 0.15 | 0.12 | 0.08 — 0.15 |
| Age | -0.02 | -0.02 — -0.01 | -0.02 | -0.02 — -0.01 |
| Smoking *(Ref: No smoking)* | -0.23 | -0.35 — -0.10 | -0.23 | -0.35 — -0.10 |
| Drinking *(Ref: No drinking)* | 0.05 | 0.04 — 0.07 | 0.05 | 0.04 — 0.07 |
| SES | 0.00 | 0.00 — 0.00 | 0.00 | 0.00 — 0.00 |
| R-square | 0.17 |  | 0.12 |  |
| F | 197.25 |  | 115.36 |  |
| N | 8654 |  | 7941.00 |  |

Note: 95% bootstrap confidence intervals are reported
